# Associations between blood inflammatory markers and bone mineral density and strength in the femoral neck: findings from the MIDUS II study

**DOI:** 10.1038/s41598-023-37377-6

**Published:** 2023-07-01

**Authors:** Zixi Huang, Zhenyan Xu, Rong Wan, Dongxia Hu, Ying Huang

**Affiliations:** 1 Rehabilitation Department, The Second Affiliated Hospital of Nanchang University, Nanchang, 330006 Jiangxi China; 2 Cardiovascular Medicine, The Second Affiliated Hospital of Nanchang University, Nanchang, 330006 Jiangxi China; 3 Jiangxi Key Laboratory of Molecular Medicine, The Second Affiliated Hospital of Nanchang University, Nanchang, 330006 Jiangxi China; 4 Department of General Medicine, The Second Affiliated Hospital of Nanchang University, Nanchang, 330006 Jiangxi China

**Keywords:** Biochemistry, Biomarkers, Risk factors

## Abstract

Chronic and systematic inflammation have been related to increased risks of osteopenia and related fracture. However, studies concerning the association between low-grade inflammation and the bone mineral density (BMD) and strength of the femoral neck are still few and inconsistent. This study aimed to examine the relationships between blood inflammatory biomarkers and BMD and femoral neck strength in an adult-based cohort. We retrospectively analyzed a total of 767 participants included in the Midlife in the United States (MIDUS) study. The blood levels of inflammatory markers, including interleukin-6 (IL6), soluble IL-6 receptor, IL-8, IL-10, TNF-α and C-reactive protein (CRP), in these participants were measured, and their associations with the BMD and strength of the femoral neck were determined. We analyzed these 767 subjects with data concerning the BMD, bending strength index (BSI), compressive strength index (CSI), and impact strength index (ISI) in the femoral neck and inflammatory biomarkers. Importantly, our results suggest that strongly negative associations exist between the blood soluble IL6 receptor levels and the BMD (per SD change, Sβ = −0.15; P < 0.001), CSI (per SD change, Sβ = −0.07; P = 0.039), BSI (per SD change, Sβ = −0.07; P = 0.026), and ISI (per SD change, Sβ = −0.12; P < 0.001) in the femoral neck after adjusting for age, gender, smoked cigarettes regularly, number of years drinking, BMI and regular exercise. However, the inflammatory biomarkers, including blood IL-6 (per SD change, Sβ = 0.00; P = 0.893), IL-8 (per SD change, Sβ = −0.00; P = 0.950), IL-10 (per SD change, Sβ = −0.01; P = 0.854), TNF-α (per SD change, Sβ = 0.04; P = 0.260) and CRP (per SD change, Sβ = 0.05; P = 0.137), were not strongly associated with the BMD in the femoral neck under the same conditions. Similarly, there was no significant difference in the relationships between the inflammatory biomarkers (IL-6, IL-8, IL-10, TNF-α and CRP) and the CSI, BSI, and ISI in the femoral neck. Interestingly, in concomitant inflammation-related chronic diseases, only arthritis affected the soluble IL-6 receptor and the CIS (interaction P = 0.030) and SIS (interaction P = 0.050) in the femoral neck. In this cross-sectional analysis, we only observed that high blood levels of soluble IL-6 receptor were strongly associated with reduced BMD and bone strength in the femoral neck. The independent associations between the other inflammatory indicators, including IL-6, IL-8, IL-10, TNF-α and CRP, and the BMD and femoral neck strength in an adult-based cohort were not significant.

## Introduction

A progressive increase in chronic and systemic inflammatory conditions is a main characteristic of the process of aging^1,2^, which is accompanied by a two- to fourfold increase in the circulating levels of inflammatory biomarkers^3,4^, linking the process to large ranges of common health problems, including bone loss and related fractures^5–7^.

In basic research, several studies have suggested that inflammatory cytokines can alter bone remodeling by promoting the expression of macrophage-colony stimulating factor (M-CSF) and the nuclear factor kappa B signaling pathway and inhibit osteoprotegerin production in vitro and in vivo^8–13^. Among clinical studies, prospective and retrospective studies have also increasingly shown that the increased circulating levels of various proinflammatory cytokines are strongly related to accelerated bone loss and a lower bone mineral density (BMD) and contribute to a higher fracture risk in older adults^5,6,14,15^. However, in recent years, some other clinical evidence failed to find a significant association between inflammatory cytokines and the BMD or fracture risk^16–18^. For example, the OsteoLaus study involving 1390 postmenopausal women reported that bone structure parameters and bone imaging are not associated with low-grade cytokine levels (within the normal range)^16^. The authors did not observe a relationship between prevalent or incident fractures and high-sensitivity C-reactive protein (hs-CRP) or associations between the blood interleukin (IL)-1β, IL-6 and tumor necrosis factor (TNF-α) levels and the BMD or fractures^16^. The Framingham Offspring Study (1996–2001) reported that the serum inflammatory biomarker concentrations, including IL-6, CRP and TNF-α, were associated with the BMD in premenopausal women but not in men. The authors finally concluded that the lack of consistency in their results suggests that elevated blood levels of inflammatory biomarkers might not be risk factors for a reduced BMD^17^. In addition, a longitudinal study with a mean follow-up time of 11.6 years (maximum 16.9 years) reported that CRP was not an indicator of a low BMD, bone loss, or fracture in elderly women^18^. These conflicting results may be related to the various health statuses, BMD positions used in the analysis and age groups in the different study populations^16–18^. For instance, concomitant inflammation related to pathologies or chronic diseases, such as insulin resistance, atherosclerosis, vascular disease, chronic obstructive pulmonary disease (COPD), Parkinson’s disease, dementia and others^19^, may be important confounders leading to inconsistent conclusions.

In the present study, we sought to replicate these findings concerning the relationship between blood inflammatory biomarkers (IL-6, soluble IL-6 receptor, IL-8, IL-10, TNF-α and CRP) and the BMD and bone strength in the femoral neck using a larger and more representative sample of 1255 participants who participated in a blood biomarker project in the Midlife in the United States (MIDUS) II study. We speculated that proinflammatory cytokines, including IL-6, soluble IL-6 receptor, IL-8, TNF-α and CRP, and anti-inflammatory cytokines, including IL-10, were inversely or positively correlated with the BMD or strength of the femoral neck. By retrospectively assessing blood biomarkers, we can comprehensively and convincingly confirm these relationships.

## Methods

### Study samples

**Figure 1:**
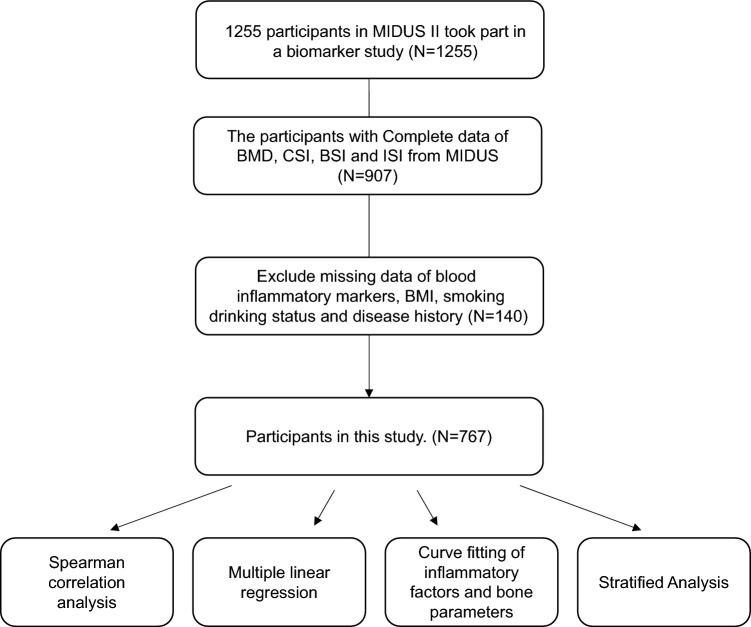
Flow chart of the subject inclusion.

We retrospectively analyzed the MIDUS study, which was conducted in 1995 and included 7108 noninstitutionalized adults, and 4,963 subjects participated in the first follow-up in MIDUS II from 2004 to 2006^20,21^. During this follow-up period, 1255 adults participated in a biomarker project involving blood inflammation biomarkers, physical examinations and medical history data^22^. After each participant volunteered to visit the clinical research centers (the Georgetown University, the University of California-Los Angeles or the University of Wisconsin), trained staff or clinicians performed a fasting blood draw and a collection of medical history, and conducted a physical examination. Of the samples participating in the biomarker project (N = 1225), in total, 907 participants had complete BMD, bending strength index (BSI), compressive strength index (CSI), and impact strength index (ISI) data. After further excluding analysis variables in the present study, including blood inflammatory markers, body mass index (BMI), smoking, drinking status and concomitant diseases (N = 140), 767 study samples were finally enrolled in our study as shown in Fig. 1.

### Blood inflammatory biomarkers

As described in the MIDUS documents from ICPSR (www.icpsr.umich.edu/web/pages/ICPSR/), fasting blood samples were collected from all participants before breakfast and then further processed and analyzed in the MIDUS Biocore Lab. The blood inflammatory biomarkers, including IL-6, IL-8, IL-10, TNF-α and CRP are described in detail in the MIDUS documents in ICPSR^21,22^.

### Assessment of BMD and bone strength in the femoral neck

The BMD values of the left hip in each participant were measured by dual-energy X ray absorptiometry (DXA) scans. The femoral neck width (FNW) and femoral neck axis length (FNAL) were measured from the hip scans according to the manufacturer’s guidelines. Composite indices of the femoral neck strength, including the CSI, BSI, and ISI, were calculated. These three bone strength indices were recorded in grams per kilogram meter (g/kg m).

### Covariates

The following covariates were included: age (years), sex (male and female), BMI, smoking status, number of drinking years, regular exercise and medical history [ever arthritis, ever transient cerebral ischemia (TIA) or stroke, ever diabetes and ever cancer]. The smoking status was defined as “whether or not ever smoked cigarettes regularly”. Regular exercise was defined as “whether or not engage in regular exercise or activity of any type for 20 min or more at least 3 times/week”. In addition to sex, all categorical variables, including the smoking status, regular exercise and medical history, were classified as “yes” or “no”. The BMI equals weight (kg) divided by the square of height (cm).

### Statistical analysis

In the current study, the statistical analyses were conducted by EmpowerStats 3.0. A P value ≤ 0.05 was considered significant. The distributions of the variables were examined by Kolmogorov–Smirnov test. The continuous variables are expressed as the median [interquartile range (IQR)], and the categorical variables are presented as n (%). Spearman and scatter plot analyses were used to assess the association between blood inflammatory biomarkers and the BMD, CSI, BSI, and ISI of the femoral neck. Then, these continuous variables with non-normal distribution were further standardized and regression models were used to analyze the independent effect of blood inflammatory biomarkers on adverse changes in the BMD, CSI, BSI, and ISI of the femoral neck.

In the calibration analysis, six types of blood inflammatory biomarkers were considered independent predictors of adverse changes in the BMD, CSI, BSI, and ISI in the femoral neck. In Model 1, each inflammatory biomarker (IL-6, soluble IL-6 receptor, IL-8, IL-10, TNF-α and CRP) was regressed on the BMD, CSI, BSI, and ISI in the femoral neck with covariates, and age and gender were adjusted. Model 2 adjusted for age, gender, ever smoked cigarettes regularly and number of drinking years. Model 3 adds BMI and regular exercise as covariates to Model 2. Specifically, subgroup analyses were further used to evaluate the effects of medical history, including ever arthritis, ever transient cerebral ischemia (TIA) or stroke, ever diabetes and ever cancer, on the relationships between the blood levels of inflammatory biomarkers and the BMD, CSI, BSI, and ISI of the femoral neck. Similar to the Model 3 analysis, age, sex, ever smoked cigarettes regularly, number of drinking years, BMI and regular exercise were adjusted in each subgroup analysis.

### Ethics approval and consent to participate

All methods were carried out in accordance with relevant guidelines and regulations (the Declaration of Helsinki), approval from the institutional review board was obtained from each MIDUS center of the MIDUS II Biomarker Project, and all study participants provided written consent. We freely obtained these data from ICPSR (https://www.icpsr.umich.edu/web/pages/ICPSR/) where MIDUS documents is stored for free use.

## Results

### Characteristics of the participants

We analyzed 767 subjects with data concerning the BMD, CSI, BSI, and ISI in the femoral neck and inflammatory biomarkers. The demographic information, including age, sex, ever smoked cigarettes regularly, number of years drinking, BMI and regular exercise, of these subjects is described in Table 1. Among these study samples, the values of the BMD, CSI, BSI, and ISI in the femoral neck were 0.89 (0.77–1.02) gms/cm^2^, 3.53 (3.08–4.07), 1.19 (1.03–1.37) g/kg m and 0.20 (0.17–0.23) g/kg m, respectively. The blood inflammatory markers, including IL-6, soluble IL-6 receptor, IL-8, IL-10, TNF-α and CRP, were 0.80 (0.54–1.19) pg/mL, 33,153.00 (26,479.50–41,117.00) pg/mL, 12.49 (9.38–15.83) pg/mL, 0.21 (0.16–0.31) pg/mL, 1.99 (1.63–2.39) pg/mL and 1.41 (0.68–3.37) μg/mL, respectively.

**Table 1:** Characteristics for study subjects (n = 767).

Variables	N (%) or median (interquartile range)
Age (year)	52 (44–60)
Gender (male), n (%)	326 (42.50%)
BMI (kg/m2)	28.43 (25.00–32.89)
Ever smoked cigarettes regularly, n (%)	400 (52.15%)
Number of years drank that much (year)	5 (2–15)
regular exercise, n (%)	583 (76.01%)
Medical history	
Ever had arthritis, n (%)	333 (43.42%)
Ever had stroke, n (%)	28 (3.65%)
Ever had diabetes, n (%)	90 (11.73%)
Ever had cancer, n (%)	102 (13.30%)
BMD and bone strength	
Femoral neck BMD (g/cm^2^)	0.89 (0.77–1.02)
Femoral neck CSI (g/kg m)	3.53 (3.08–4.07)
Femoral neck BSI (g/kg m)	1.19 (1.03–1.37)
Femoral neck ISI (g/kg m)	0.20 (0.17–0.23)
Blood inflammatory markers	
IL6 (pg/mL)	0.80 (0.54–1.19)
IL8 (pg/mL)	12.49 (9.38–15.83)
IL10 (pg/mL)	0.21 (0.16–0.31)
TNF-α (pg/mL)	1.99 (1.63–2.39)
Soluble IL6 receptor (pg/mL)	33,153.00 (26,479.50–41,117.00)
CRP (μg/mL)	1.41 (0.68–3.37)

*BMI* body mass index, *BMD* bone mineral density, *CSI* compression strength index, *BSI* bending strength index, *ISI* impact strength index, *MSD* meso scale discovery, *IL* interleukin, *TNF-α* tumor necrosis factor-α, *CRP* C-reactive protein.

### Relationships between blood inflammatory biomarkers and the BMD and bone strength (CSI, BSI, and ISI) in the femoral neck

**Figure 2:**
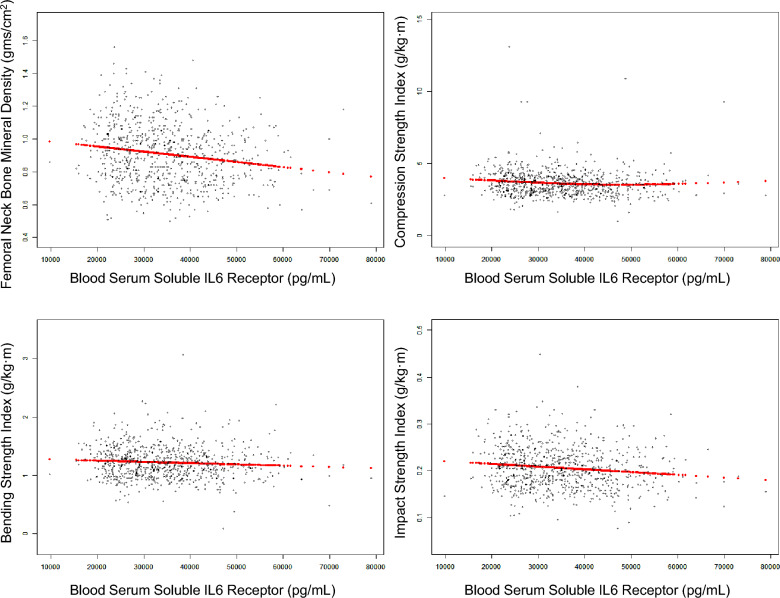
Scatter diagram of the associations between the blood soluble IL-6 receptor levels and lower BMD and bone strength.

As shown in Supplementary materials 1, Spearman’s methods demonstrated that the blood levels of IL-6, soluble IL-6 receptor and CRP, but not those of IL-8, IL-10 and TNF-α, are inversely related to the BMD, CSI, BSI and ISI in the femoral neck (all P < 0.05). The scatter plot analysis suggested that the elevated blood levels of soluble IL-6 receptor were closely associated with reduced values of the BMD, CSI, BSI and ISI (Fig. 2, all < 0.05).

As shown in Table 2, Model 1 suggested that significant inverse associations exist between the blood soluble IL-6 receptor levels and the BMD (per SD change, Sβ = − 0.15; 95% CI −0.22, −0.08; P < 0.001), CSI (per SD change, Sβ = −0.08; 95% CI −0.15, −0.01; P = 0.029), BSI (per SD change, Sβ = −0.07; 95% CI −0.14, −0.01; P = 0.035), and ISI (per SD change, Sβ = −0.13; 95% CI -0.20, -0.06; P < 0.001) in the femoral neck after controlling for age and sex. Importantly, Model 3 still suggested that strong negative associations exist between the blood soluble IL-6 receptor levels and the BMD (per SD change, Sβ = −0.15; 95% CI −0.22, −0.09; P < 0.001), CSI (per SD change, Sβ = −0.07; 95% CI −0.13, 0.00); P = 0.039), BSI (per SD change, Sβ = −0.07; 95% CI −0.13, −0.01; P = 0.026), and ISI (per SD change, Sβ = −0.12; 95% CI −0.18, −0.06; P < 0.001) in the femoral neck after further adjusting for smoked cigarettes regularly; the number of years drinking, BMI and regular exercise. However, our results did not show that inflammatory biomarkers, including blood IL-6 (per SD change, Sβ = 0.00; 95% CI -0.06, 0.07; P = 0.893), IL-8 (per SD change, Sβ = −0.00; 95% CI −0.06, 0.06; P = 0.950), IL-10 (per SD change, Sβ = −0.01; 95% CI −0.07, 0.06; P = 0.854), TNF-α (per SD change, Sβ = 0.04; 95% CI −0.03, 0.10; P = 0.260) and CRP (per SD change, Sβ = 0.05; 95% CI −0.02, 0.12; P = 0.137), were strongly associated with the BMD in the femoral neck after controlling for age, sex, ever smoked cigarettes regularly, number of years drinking, BMI and regular exercise. Similarly, there was no significant difference in the relationships between the inflammatory biomarkers (IL-6, IL-8, IL-10, TNF-α and CRP) and the CSI, BSI, and ISI in the femoral neck.

**Table 2:** Linear regression analysis for relationships between blood inflammatory markers level and BMD and bone strength in femoral neck.

Variables	Model 1		Model 2		Model 3	
	Per SD change (95% CI)	*P* value	Per SD change (95% CI)	*P* value	Per SD change (95% CI)	*P* value
Femoral neck BMD (g/cm^2^)						
IL6 (pg/mL)	0.04 (−0.03, 0.11)	0.233	0.04 (−0.03, 0.10)	0.272	0.00 (−0.06, 0.07)	0.893
IL8 (pg/mL)	−0.02 (−0.08, 0.05)	0.659	−0.02 (−0.09, 0.05)	0.563	−0.00 (−0.06, 0.06)	0.950
IL10 (pg/mL)	−0.01 (−0.08, 0.05)	0.705	−0.01 (−0.08, 0.06)	0.733	−0.01 (−0.07, 0.06)	0.854
TNF-α (pg/mL)	0.08 (0.01, 0.15)	0.025	0.08 (0.01, 0.15)	0.024	0.04 (−0.03, 0.10)	0.260
Soluble IL6 receptor (pg/mL)	−0.15 (−0.22, −0.08)	< 0.001	−0.14 (−0.21, −0.08)	< 0.001	−0.15 (−0.21, −0.09)	< 0.001
CRP (μg/mL)	0.17 (0.11, 0.24)	< 0.001	0.17 (0.11, 0.24)	< 0.001	0.05 (−0.02, 0.12)	0.137
CSI (g/kg m)						
IL6 (pg/mL)	−0.05 (−0.12, 0.02)	0.166	−0.05 (−0.13, 0.02)	0.129	−0.01 (−0.08, 0.05)	0.654
IL8 (pg/mL)	0.03 (−0.04, 0.10)	0.459	0.02 (−0.05, 0.09)	0.558	−0.00 (−0.06, 0.06)	0.969
IL10 (pg/mL)	−0.01 (−0.08, 0.06)	0.776	−0.01 (−0.08, 0.06)	0.815	−0.02 (−0.08, 0.05)	0.638
TNF-α (pg/mL)	−0.04 (−0.12, 0.03)	0.232	−0.04 (−0.12, 0.03)	0.232	0.01 (−0.06, 0.07)	0.810
Soluble IL6 receptor (pg/mL)	−0.08 (−0.15, −0.01)	0.029	−0.07 (−0.14, 0.00)	0.050	−0.07 (−0.13, 0.00)	0.039
CRP (μg/mL)	−0.13 (−0.21, −0.06)	< 0.001	−0.13 (−0.21, −0.06)	< 0.001	0.02 (−0.05, 0.09)	0.570
BSI (g/kg m)						
IL6 (pg/mL)	−0.04 (−0.11, 0.03)	0.295	−0.04 (−0.11, 0.03)	0.286	0.01 (−0.06, 0.07)	0.869
IL8 (pg/mL)	0.01 (−0.06, 0.08)	0.730	0.01 (−0.06, 0.08)	0.797	−0.01 (−0.08, 0.05)	0.633
IL10 (pg/mL)	−0.01 (−0.08, 0.06)	0.859	−0.01 (−0.08, 0.06)	0.848	−0.01 (−0.08, 0.05)	0.653
TNF-α (pg/mL)	−0.04 (−0.11, 0.03)	0.260	−0.04 (−0.11, 0.03)	0.263	0.01 (−0.05, 0.08)	0.640
Soluble IL6 receptor (pg/mL)	−0.07 (−0.14, −0.01)	0.035	−0.07 (−0.14, −0.00)	0.040	−0.07 (−0.13, −0.01)	0.026
CRP (μg/mL)	−0.14 (−0.21, −0.07)	< 0.001	−0.14 (−0.21, −0.07)	< 0.001	0.03 (−0.04, 0.09)	0.376
ISI (g/kg m)						
IL6 (pg/mL)	−0.07 (−0.14, −0.00)	0.045	−0.07 (−0.14, −0.00)	0.038	−0.02 (−0.08, 0.03)	0.410
IL8 (pg/mL)	0.04 (−0.03, 0.11)	0.296	0.03 (−0.04, 0.10)	0.367	0.00 (−0.05, 0.06)	0.866
IL10 (pg/mL)	−0.01 (−0.08, 0.06)	0.722	−0.01 (−0.08, 0.06)	0.727	−0.02 (−0.08, 0.04)	0.477
TNF-α (pg/mL)	−0.04 (−0.11, 0.03)	0.286	−0.04 (−0.11, 0.03)	0.289	0.03 (−0.03, 0.08)	0.397
Soluble IL6 receptor (pg/mL)	−0.13 (−0.20, −0.06)	< 0.001	−0.13 (−0.20, −0.06)	< 0.001	−0.12 (−0.18, −0.06)	< 0.001
CRP (μg/mL)	−0.16 (−0.23, −0.09)	< 0.001	−0.16 (−0.23, −0.09)	< 0.001	0.03 (−0.03, 0.10)	0.272

Model 1: adjusted for age and gender.

Model 2: adjusted for age, gender, ever smoked cigarettes regularly and number of years drank that much.

Model 3: adjusted for age, gender, ever smoked cigarettes regularly, number of years drank that much, BMI and regular exercise.

*BMD* bone mineral density, *CSI* compression strength index, *BSI* bending strength index, *ISI* impact strength index, *IL* interleukin, *TNF* tumor necrosis factor, *CRP* C-reactive protein, *BMI* body mass index.

### Arthritis differences in the associations between the soluble IL-6 receptor and bone strength in the femoral neck

Concomitant inflammation-related chronic diseases, such as cancer, vascular disease, diabetes and arthritis, have been confirmed to be associated with systemic inflammation in the body^19^. Our subgroup analysis showed that arthritis can affect the associations between the soluble IL-6 receptor and the CIS (interaction P = 0.030) and ISI (interaction P = 0.050) in the femoral neck. The elevated soluble IL-6 receptor levels were independently and negatively associated with a reduced CIS and ISI in the subjects (all P < 0.05) without arthritis but not in those with arthritis (all P > 0.05; Table 3). However, “ever had transient cerebral ischemia (TIA) or stroke”, “ever had diabetes” and “ever had cancer”, as well as gender and BMI (Supplementary materials 1), did not affect the relationships between the blood levels of inflammatory biomarkers and the BMD (all interaction P >0.05), CSI (all interaction P > 0.05), BSI (all interaction P > 0.05), and ISI (all interaction P > 0.05) in the femoral neck.

**Table 3:** Subgroup analysis based medical history for relationships between blood soluble IL6 receptor levels and BMD and bone strength in femoral neck.

Variables	Per SD change (95% CI)	*P* value	Interaction P
Femoral neck BMD (g/cm^2^)			
Ever had arthritis	−0.12 (−0.21, −0.03)	0.008	0.348
NO	−0.18 (−0.26, −0.09)	< 0.001	
Ever had stroke	−0.08 (−0.46, 0.30)	0.696	0.726
NO	−0.15 (−0.22, −0.09)	< 0.001	
Ever had diabetes	−0.18 (−0.37, 0.00)	0.058	0.525
NO	−0.14 (−0.20, −0.07)	< 0.001	
Ever had cancer	−0.04 (−0.22, 0.15)	0.699	0.199
NO	−0.16 (−0.23, −0.09)	< 0.001	
CSI (g/kg m)			
Ever had arthritis	0.01 (−0.08, 0.09)	0.911	0.030
NO	−0.13 (−0.22, −0.05)	0.003	
Ever had stroke	−0.18 (−0.63, 0.28)	0.454	0.638
NO	−0.07 (−0.13, −0.01)	0.035	
Ever had diabetes	−0.14 (−0.32, 0.03)	0.108	0.409
NO	−0.06 (−0.13, 0.01)	0.098	
Ever had cancer	−0.03 (−0.19, 0.13)	0.735	0.654
NO	−0.07 (−0.14, −0.00)	0.042	
BSI (g/kg m)			
Ever had arthritis	−0.03 (−0.13, 0.06)	0.479	0.236
NO	−0.11 (−0.19, −0.03)	0.008	
Ever had stroke	−0.34 (−1.12, 0.43)	0.394	0.206
NO	−0.07 (−0.13, −0.01)	0.019	
Ever had diabetes	−0.11 (−0.27, 0.05)	0.184	0.728
NO	−0.07 (−0.14, −0.00)	0.037	
Ever had cancer	−0.01 (−0.22, 0.20)	0.913	0.458
NO	−0.08 (−0.15, −0.02)	0.011	
ISI (g/kg m)			
Ever had arthritis	−0.07 (−0.16, 0.02)	0.117	0.050
NO	−0.17 (−0.25, −0.10)	< 0.001	
Ever had stroke	−0.15 (−0.72, 0.42)	0.615	0.915
NO	−0.13 (−0.18, −0.07)	< 0.001	
Ever had diabetes	−0.15 (−0.31, 0.02)	0.087	0.828
NO	−0.12 (−0.19, −0.06)	< 0.001	
Ever had cancer	−0.03 (−0.22, 0.16)	0.790	0.212
NO	−0.14 (−0.20, −0.08)		

Adjusted for age, gender, ever smoked cigarettes regularly, number of years drank that much, BMI and regular exercise.

*BMD* bone mineral density, *CSI* compression strength index, *BSI* bending strength index, *ISI* impact strength index, *IL* interleukin.

## Discussion

Using data from the MIDUS II study, which included a cohort of 1,255 noninstitutionalized adults, we verified the associations between blood inflammatory biomarker levels and the BMD and bone strength (CSI, BSI and ISI) in the femoral neck. Higher blood soluble IL-6 receptor levels were associated with a lower BMD and bone strength in the femoral neck. The fully adjusted relationship was slightly altered after adjusting for many covariates (age, sex, ever smoked cigarettes regularly, number of years drinking, BMI and regular exercise). There were no significant differences between blood inflammatory biomarkers (IL-6, IL-8, IL-10 and TNF-α) and the BMD and bone strength in the femoral neck. Immune inflammatory reactions change across the course of life, which has been considered attributable to overloading the immune system in response to persistent low-grade inflammation and stress^24,25^. Existing evidence has demonstrated that low-level inflammation tends to cause DNA damage, cellular senescence and biological aging. Studies have even demonstrated the promotional effect of proinflammatory cytokines, including IL-1β, IL-6 and TNF-α, in altering bone structure^26^ by inhibiting the activation and proliferation of osteoblasts. From a clinical perspective, previous studies have also reported various associations between IL-6, IL-8, IL-10, CRP and TNF-α and BMD and fracture risk^27^.

### Relationship between blood CRP levels and the BMD and bone strength of the femoral neck

CRP is a very sensitive and widely studied inflammatory marker. Some investigations have suggested that high hs-CRP is linked to increased risks of a lower BMD and fracture^28–30^. The Geelong Osteoporosis study reported that as CRP increased with each SD, fracture risk was significantly and independently increased by 24–32%^29^. The Rotterdam study suggested that hs-CRP was related to an increased risk of incident fracture, but this relationship was statistically significant only in females^31^. Consistently, our results also demonstrated that higher CRP levels were related to lower BMD and bone strength in the femoral neck, and this relationship was dependent on age, sex, ever smoked cigarettes regularly and number of years drinking. However, some other studies were unable to find significant relationships^29,31,32^. Interestingly, continued adjustment for BMI and regular exercise completely eliminated this independent association in our study. Here, we observed that the demographic characteristics, lifestyle and disease history of the study sample may be important confounding factors, resulting in differences in numerous previous studies.

### Association between the blood soluble IL-6 receptor levels and the BMD and bone strength of the femoral neck

IL-6, characterized as a cytokine with multiple functions, plays an important role in regulating immunologic and inflammatory responses in vivo and is involved in regulating bone resorption and osteoclastogenesis in some pathological conditions. IL-6 can play a biological role by binding a signal transducing receptor (gp130) and soluble receptor (sIL-6R)^33^. Interestingly, the existing evidence concerning IL-6 is also controversial. On the one hand, a previous study reported that higher blood IL-6 levels were positively related to a reduced BMD^6^. On the other hand, Cauley et al. also observed elevated IL-6 levels in males, but not in females, who experienced hip fractures^14,34^, which is not consistent with our results that higher blood soluble IL-6 receptor, but not blood IL-6 levels, were related to lower BMD and bone strength in the femoral neck after fully adjusting for covariates. In addition to the different results caused by the confounding factors described above, the measurement repeatability of blood IL-6 is relatively difficult, which could partly explain the discrepancies in the previous literature. Furthermore, regarding the pathological mechanism, blood IL-6 is the only cell factor with daily fluctuation, and its levels were at the lowest point in the morning^35^. Thus, the time of blood drawing may also be a factor leading to differences among these research conclusions. However, a recent study also surprisingly concluded that only blood sIL-6R, but not IL-6, was a significant biomarker associated with BMD changes in postmenopausal women^33^, and these results are similar to our study. The unique difference was that our results did not show a significant sex difference in the independent association.

### Relationships between the blood TNF-α levels and the BMD and bone strength of the femoral neck

TNF-α, which originates from activated macrophages, is also an important cytokine for regulating bone resorption and osteoclastogenesis when inflammation occurs in vitro and in vivo. The Health Aging and Body Composition Study involving thousands of individuals (> 70 years) demonstrated that the fracture incidence was significantly elevated with elevated TNF-α levels^14^. However, chronic inflammatory diseases were not assessed in the patients, and the results were not adjusted for lifestyle factors, such as BMI and regular exercise. Inconsistently, there was no significant difference in the relationships between TNF-α and bone strength (CSI, BSI, and ISI) in the femoral neck in our study, even if it was only adjusted for age and sex. The short half-life of blood TNF-α concentrations may partly explain the discrepancy because elevated TNF-α levels are usually transient^14^. Therefore, the time point of blood measurement may also be an important confounding factor responsible for the discrepancy.

### Relationships between the blood IL-8 levels and the BMD and bone strength of the femoral neck

IL-8, a cytokine belonging to the chemokine family, has a cellular chemotactic effect on neutrophils to regulate their inflammatory response. A previous study reported an association between elevated IL-8 and an increased decline of the lumbar spine^36^, which is consistent with the adverse effects of inflammatory responses on the BMD. However, this conclusion was unreliable because there was not analysis in the fully adjusted model. In our study, we did not confirm that an independent association exists between the blood levels of IL-18 and the BMD and bone strength in the femoral neck after adequately controlling for confounding factors, including age, sex, ever smoked cigarettes regularly, number of years drinking, BMI and regular exercise.

### Relationships between the blood IL-10 levels and the BMD and bone strength of the femoral neck

As an anti-inflammatory cytokine, IL-10 can dampen immune activation stimulated by lipopolysaccharide and T helper 1-cell activity. Experiments have shown that IL-10 deficiency can cause adverse outcomes in bone, such as attenuating bone formation and progressing osteopenia in animal models^37^. A previous human study also supported the cellular effect based on findings suggesting that higher variants of IL-10 was associated with a reduced BMD in postmenopausal females^38^. Inconsistently, in the present study, we also identified associations between the blood levels of IL-10 and the BMD and bone strength in the femoral neck, reporting no significant differences, even if no confounding factor was adjusted.

### Strengths and limitations

Currently, to the best of our knowledge, this study represents the first clinical exploration of the associations between bone parameters (BMD, CSI, BSI, and ISI) in the femoral neck and inflammatory biomarkers (IL-6, soluble IL-6 receptor, IL-8, IL-10, CRP and TNF-α) within the normal range among large population-based noninstitutionalized adults. We observed for the first time that higher soluble blood IL-6 receptor levels were associated with an increased risk of bone loss (reduced BMD) and decreased bone strength (reduced CSI, BSI, and ISI) in the femoral neck. Importantly, we should also emphasize several limitations. First, we retrospectively conducted a cross-sectional analysis, and circulating inflammatory cytokines were measured once during a certain period. Data concerning the dynamic changes in blood inflammatory cytokines are not available. Additionally, the highest inflammatory mediator concentrations should appear in the bone marrow microenvironment rather than circulating blood. Second, although age, sex, ever smoked cigarettes regularly, number of years drinking, BMI and regular exercise were adjusted in our study, medication use such as steroid and non-steroidal anti-inflammatory drugs, calcium carbonate and others was not analyzed in our study, which may affect the stability of our results to some extent. Third, we only measured sIL-6R, which is more stable than IL-6, and we did not measure the receptor levels of the other inflammatory factors. We still do not know why we only observed an association of IL-6 receptor levels, but not the other inflammatory markers, with bone health parameters, which needs research evidence in the future. Fourth, the medical history (ever arthritis, ever TIA or stroke, ever diabetes and ever cancer) used was derived from the participants' self-reports; thus, the precise medical history of these subjects was unknown, potentially impacting the results. Fifth, only 767 study subjects were enrolled from the biomarker project of the MIDUS II study. The remaining subjects with incomplete data were not analyzed in our study (N = 488). Additionally, there are no variables such as race, lumbar BMD included in this study, which may slightly hinder the external implementation of the conclusions.

## Conclusions

We observed significant associations between the blood soluble IL-6 receptor, but not the other inflammatory biomarkers (IL-6, IL-8, IL-10, CRP and TNF-α), and the BMD and bone strength of the femoral neck, adding new evidence to the previous theory that femoral neck health can be influenced by the inflammatory phenotype.

## Supplementary Information

Supplementary Information.

## Data Availability

The datasets generated and/or analysed during the current study are available in the [ICPSR] repository (https://www.icpsr.umich.edu/web/pages/ICPSR/).
