# Supplementary material for: Associations between blood inflammatory markers and bone mineral density and strength in the femoral neck: findings from the MIDUS II study
Source: Sci Rep. 2023 Jul 1;13:10662. doi: 10.1038/s41598-023-37377-6 (PMC10314938; doi:10.1038/s41598-023-37377-6)
Supplement: Supplementary file 1 — Supplementary Information. [file 41598_2023_37377_MOESM1_ESM.docx]

| **Supplementary materials 1.** methods of Spearman for correlation between blood inflammatory markers levels and BMD and bone strength in femoral neck. | | | | |
| --- | --- | --- | --- | --- |
| **Variables** | Femoral Neck BMD (gms/cm^2^) | CSI (g/kg·m) | BSI (g/kg·m) | ISI (g/kg·m) |
| IL6 (pg/mL) | 0.230** | -0.170** | -0.150** | -0.182** |
| IL8 (pg/mL) | -0.034 | 0.053 | 0.025 | 0.045 |
| IL10 (pg/mL) | 0.058 | -0.005 | -0.028 | -0.006 |
| TNF-α (pg/mL) | 0.009 | -0.104** | -0.081* | -0.069 |
| Soluble IL6 receptor (pg/mL) | -0.178** | -0.127** | -0.081* | -0.132** |
| CRP (ug/mL) | 0.208** | -0.259** | -0.245** | -0.304** |
| Spearman correlation analysis，*<0.05; **<0.01.  BMD: Bone Mineral Density; CSI: Compression Strength Index; BSI: Bending Strength Index; ISI: Impact Strength Index; MSD: Meso Scale Discovery; IL: Interleukin; TNF: Tumor Necrosis Factor; CRP: C-Reactive Protein. | | | | |

| **Supplementary materials 2：**Correlation between blood soluble IL6 receptor and BMD and bone strength in femoral neck. | | | |
| --- | --- | --- | --- |
| **Variables** | **perSD change (95% CI)** | ***P* Value** | **Interaction P** |
| **Femoral Neck BMD (gms/cm^2^)** | |  |  |
| Male | -0.14 (-0.24, -0.03) | 0.012 | 0.708 |
| Female | -0.15 (-0.23, -0.08) | <0.001 |  |
| BMI≤24 (kg/m2) | -0.05 (-0.19, 0.09) | 0.476 | 0.197 |
| BMI＞24 (kg/m2) | -0.17 (-0.24, -0.09) | <0.001 |  |
| **CSI (g/kg·m)** |  |  |  |
| Male | -0.09 (-0.18, -0.01) | 0.035 | 0.586 |
| Female | -0.05 (-0.14, 0.04) | 0.260 |  |
| BMI≤24 | -0.07 (-0.20, 0.06) | 0.290 | 0.931 |
| BMI＞24 | -0.06 (-0.14, 0.02) | 0.117 |  |
| **BSI (g/kg·m)** |  |  |  |
| Male | -0.07 (-0.16, 0.03) | 0.171 | 0.991 |
| Female | -0.07 (-0.15, 0.00) | 0.050 |  |
| BMI≤24 | -0.02 (-0.18, 0.13) | 0.757 | 0.571 |
| BMI＞24 | -0.08 (-0.15, -0.00) | 0.045 |  |
| **ISI (g/kg·m)** |  |  |  |
| Male | -0.10 (-0.20, -0.00) | 0.050 | 0.554 |
| Female | -0.14 (-0.21, -0.07) | <0.001 |  |
| BMI≤24 | -0.11 (-0.27, 0.04) | 0.148 | 0.973 |
| BMI＞24 | -0.12 (-0.19, -0.05) | 0.001 |  |
| Adjusted for Age, Gender, Ever smoked cigarettes regularly, Number of years drank that much, BMI and regular exercise.  BMD: Bone Mineral Density; CSI: Compression Strength Index; BSI: Bending Strength Index; ISI: Impact Strength Index; IL: Interleukin; BMI: Body Mass Index. | | | |
